# Effects of clinical nursing pathway on postoperative satisfaction and quality of life of patients with subarachnoid hemorrhage

**DOI:** 10.12669/pjms.40.4.7800

**Published:** 2024

**Authors:** Min Ge, Ning Gan, Yuchao Shan, Nan Tian

**Affiliations:** 1 Min Ge, Department of Neurosurgery, Baoding No.1 Central Hospital, Baoding 071000, Hebei, China; 2 Ning Gan, Department of Neurosurgery, Baoding No.1 Central Hospital, Baoding 071000, Hebei, China; 3 Yuchao Shan, Department of Neurosurgery, Baoding No.1 Central Hospital, Baoding 071000, Hebei, China; 4 Nan Tian, Department of Neurosurgery, Baoding No.1 Central Hospital, Baoding 071000, Hebei, China

**Keywords:** Clinical Nursing Pathway, Sub Arachnoid Hemorrhage, Satisfaction, Quality of Life

## Abstract

**Objective:**

To explore the effects of clinical nursing pathway (CNP) on the postoperative satisfaction and quality of life (QOL) of patients with subarachnoid hemorrhage (SAH).

**Methods:**

This is a retrospective study. Eighty patients with SAH admitted to Baoding No.1 Central Hospital from June 2021 to January 2023 were prospectively divided into a observation group and a control group by random numbers. The control group was given routine nursing, and the observation group was additional given CNP. The prognosis, cognitive function, QOL, self-care ability, nursing satisfaction and the incidence of complications were compared between the two groups.

**Results:**

After CNP nursing, the GCS and MMSE scores in the observation group were higher than those in the control group 14 days, one month and six months after the operation; and the difference was statistically significant (p< 0.05). Six months after the operation, the SS-QOL and Ability of daily living (ADL) scores in both groups were significantly improved compared with those before the intervention; and the improvement in the observation group was significantly better than that in the control group; and the difference was statistically significant(p<0.05). The nursing satisfaction score in the observation group was significantly higher than in the control group. The total incidence of complications in the observation group was lower than that in the control group.

**Conclusions:**

The CNP intervention in perioperative period of SAH patients has remarkable clinical effect, can improve the pertinence and efficiency of nursing, promote patients to recover as soon as possible, significantly improve the QOL of patients,and is worthy of clinical popularization.

## INTRODUCTION

Subarachnoid hemorrhage (SAH) is one of the clinical critical illness. It is a clinical syndrome caused by the rupture of blood vessels at the base or surface of the brain^1^,^2^ and the blood flow into the subarachnoid space. The pathogenesis of SAH is traumatic and non-traumatic, of which non-traumatic SAH is the most common; and aneurysmal SAH is the most common, accounting for 85% of all cases.^3^ In recent years, with the development of diagnostic methods, vascular interventional techniques and perioperative management techniques, the diagnosis and treatment of SAH have developed rapidly. Moreover, as minimally invasive surgical techniques become more minimally invasive and refined, surgery, especially minimally invasive surgery, has been increasingly used in clinical treatment of SAH.^4^

However, the prognosis of patients with SAH was still poor. The case fatality rate and disability rate are still high. Studies have pointed out that^5^,^6^, the application of personalized nursing measures for patients in clinical practice can effectively reduce the incidence of SAH complications, and is conducive to the rehabilitation of patients, which is of great significance for clinical practice, but the current clinical nursing staff is seriously insufficient, unable to meet the implementation of large-scale personalized and comprehensive nursing. On the one hand, patients with SAH have complicated conditions and are difficult to nurse; on the other hand, unintentional negligence and omission caused by heavy and trivial nursing make patients less satisfied with nursing services.

Clinical Nursing Pathway (CNP) is a nursing model of clinical pathway extension.^7^ CNP is aimed at specific patient population or diseases to make schedule, clinical treatment and nursing standardized and programmed with time as the horizontal axis and nursing means as the vertical axis. Patients are examined, treated and nursed as scheduled according to the time on the path. At present, CNP is being widely practiced in the field of health worldwide. In order to better explore the clinical effect of CNP, in this study, the patients undergoing surgical treatment for SAH in our hospital were selected as the study subjects to observe the influence of CNP on patients’ QOL and nursing satisfaction.

## METHODS

This is a retrospective study. Eighty patients with SAH admitted to Baoding No.1 Central Hospital from June 2021 to January 2023 were selected and divided into a CNP group (observation group) and a routine nursing group (control group) (n= 40, respectively) by random numbers.

### Ethical Approval

The study was approved by the Institutional Ethics Committee of Baoding No.1 Central Hospital (No.: [2022]083; August 03,2022), and written informed consent was obtained from all participants.

### Inclusion criteria:

- Patients diagnosed with SAH by imaging examination or lumbar puncture.
- Patients with non-traumatic primary SAH.
- Patients who underwent endovascular embolization or aneurysm clipping within 72 hours of onset.
- Patients with predicted survival time > 6 minutes.
- Patients with complete clinical data and follow-up data.
- Patients who were informed of this study and signed the informed consent.

### Exclusion criteria:

- Patients who received no surgical treatment.
- Patients who were complicated with intracranial tumor or extracranial injury.
- Patients with recurrent SAH.
- Patients with consciousness or mental disorders before onset.
- Patients and their family members who do not cooperate with clinical diagnosis and treatment workers.
- Patients with incomplete clinical data or patients lost to the follow-up.

### Nursing methods

Patients in the control group were treated with routine nursing intervention, including vital signs monitoring, condition observation, preoperative preparation and postoperative nursing guidance, etc. Patients in the observation group were additionally treated with CNP intervention as follows:

- A CNP intervention group was established, with members trained on the content, methods and advantages
- A CNP intervention execution table was developed; and a personalized clinical pathway intervention table was developed based on previous nursing experience and after group discussion; - re-operative nursing: stabilizing the patient’s mood, issuing health education manuals, one-to-one explanation of the necessity of SAH surgical treatment to patients, postoperative rehabilitation training methods, answering the questions of patients and families, urging patients’ families to accompany patients, and showing patients cases of good post-operative rehabilitation; **-** Intraoperative nursing: adjustment of the operating room temperature 30 min before the operation, gentle movement during transportation, paying attention to protecting the patients’ private parts, and insulation intervention for patients.
- Postoperative care: According to the patients’ tolerance and condition recovery, the corresponding personalized rehabilitation nursing plan was developed; and the value and precautions of rehabilitation training to improve the condition were introduced in detail to patients and their families.
- Post-discharge nursing: rehabilitation manuals, mainly including medication and diet guidance and family rehabilitation training methods, were issued to patients and their families; patients were instructed to review regularly; telephone follow-up and outpatient reexamination were carried out to know the recovery of patients. This manuscript conforms the Enhancing the Quality and Transparency Of health Research (EQUATOR) network guidelines.

### Observation indicators:

*Prognosis:* Glasgow Coma Scale (GCS) was used to assess patients’ state of consciousness 14 days, one month and six months after the operation; and Glasgow outcome scale (GOS) was used to assess patients’ prognosis six months after the operation. There are five grades in the GOS scale: five is normal, although with slight disability; four is slightly disabled, but patients can live independently and work under protection; three is severely disabled, patients are sober but need nursing in daily life and are unable to be independent; two is a vegetative state with only eye activity and sleep cycle; one is death.

*Cognitive function:* The mini-mental state examination (MMSE) was used to assess cognitive function 14 days, one month and six months after the operation, respectively. The MMSE includes 30 items covering orientation, memory, temporal and spatial perception, attention and numeracy, and language ability. A total of 0-30 points are scored, the higher the score, the better the cognitive function; and ≤23 is classified as post-operative cognitive dysfunction (POCD). The incidence of POCD in the two groups six months after the operation was recorded.

*Assessment of QOL and self-care ability*. The QOL of patients was assessed with SS-QOL before and after intervention, respectively, to understand the QOL of patients, including four aspects, language, role, cognition and body function, with a score of 0-20 points. The higher the score, the better the QOL.

*Nursing satisfaction survey:* The American scholar Risser Inpatient Nursing Satisfaction Scale, was used to survey the nursing satisfaction of patients in both groups at discharge. The scale includes 21 items, including three dimensions of trust relationship, professional and technical ability and education relationship. The Likert 5-grade scoring method was used to score 1-5 points from “very disagree” to “very agree”. Questions 2,3,5,7,10,13,15 and 21 are negative scoring questions. The higher the score, the higher patients’ satisfaction.

*Complications:* It included headache, vomiting, electrolyte disturbance, re-hemorrhage and hydrocephalus.

### Statistical analysis

The software SPSS22.0 was used for data analysis. The measurement data were expressed as (*χ̅*;±*S*) and tested by two independent samples t-test. The counting data were expressed as n (%). χ^2^ test was used for comparison between groups. The difference was statistically significant for *p<* 0.05.

## RESULTS

There was no significant difference in the general data of patients between the two groups (*p>* 0.05). However, there was still comparability between the two groups, (Table-I). The results showed that after CNP nursing, the GCS and MMSE scores in the observation group were higher than those in the control group 14 days, one month and six months after the operation; and the difference was statistically significant (p< 0.05). The GOS score in the observation group six months after the operation was (4.23±0.73) points, which was higher than the (4.10±0.84) points in the control group; but the difference was not statistically significant (*p>* 0.05), (Table-II).

**Table-I:** Contrastive Analysis of General Data between the Study Group and the Control Group.

Item	Observation group (n = 40)	Control group (n = 40)	t/χ^2^	P
Age (y)	56.35±10.66	55.98±9.11	0.169	0.866
Gender (male/female)	25/15	23/17	0.208	0.648
Operation conditions			0.220	0.639
Endovascular embolization	27 (67.50%)	25 (62.50%)		
Aneurysm clipping	13 (32.50%)	15 (37.50%)		
Hemorrhage position			0.879	0.348
Base of the brain	28 (70.00%)	24 (60.00%)		
Brain surface	12 (30.00%)	16 (40.00%)		
GCS score			0.453	0.797
9-11 points	15 (37.50%)	13 (32.50%)		
12-14 points	19 (47.50%)	22 (55.00%)		
15 points	6 (15.00%)	5 (12.50%)		
Amount of bleeding (ml)	20.65±3.37	20.83±3.21	0.252	0.802

**Table-II:** Contrastive Analysis of Prognosis between the Observation Group and the Control Group.

Item	Observation group (n = 40)	Control group (n = 40)	t/χ^2^	P
GCS score				
14 days after the operation			6.842	0.033
9-11 points	7 (17.50%)	6 (15.00%)		
12-14 points	20 (50.00%)	30 (75.00%)		
15 points	13 (32.50%)	4 (10.00%)		
1 month after the operation			6.541	0.038
9-11 points	3 (7.50%)	3 (7.50%)		
12-14 points	13 (32.50%)	24 (60.00%)		
15 points	24 (60.00%)	13 (32.50%)		
6 months after the operation			6.630	0.036
9-11 points	0 (0.00%)	1 (2.50%)		
12-14 points	12 (30.00%)	22 (55.00%)		
15 points	28 (70.00%)	17 (42.50%)		
GOS score	4.23±0.73	4.10±0.84	0.708	0.481

There was no significant difference in the MMSE score at admission between the two groups (*p>* 0.05). The MMSE score in the observation group 14 days, one month and one months after the operation was higher than that in the control group; and the difference was statistically significant (p< 0.05). The incidence of POCD in the observation group six months after the operation was lower than that in the control group; but the difference was not statistically significant (*p>* 0.05), (Table-III).

**Table-III:** Comparison of Cognitive Function and Incidence of POCD between the Two Groups.

Group	MMSE score (point)(*χ̅*±*S*)				Incidence of POCD [cases (%)]

	At admission	14 days after the operation	1 month after the operation	6 months after the operation	
Observation group	21.23±1.42	18.65±0.66	22.53±0.85	25.83±2.62	15 (37.50)
Control group	20.60±1.58	18.00±0.72	21.85±0.95	24.53±2.45	20 (50.00)
t value	1.858	4.215	3.357	2.292	1.270
P value	0.067	0.000	0.001	0.025	0.260

The difference in the SS-QOL and ADL scores at admission were not statistically significant (*p>* 0.05). The SS-QOL and ADL scores six months after the operation were significantly improved compared with those before the intervention; and the improvement in the observation group was significantly better than that in the control group; and the difference was statistically significant (p< 0.05), (Table-IV).

**Table-IV:** Comparison of SS-QOL and ADL Scores Between the Two Groups (*χ̅*±*S*).

Group	SS-QOL score (point)								ADL score	

	Physical function		Linguistic function		Role function		Cognitive function			

	At admission	6 months after the operation	At admission	6 months after the operation	At admission	6 months after the operation	At admission	6 months after the operation	At admission	6 months after the operation
Observation group	4.75±0.44	10.90±0.90	5.90±0.71	14.98±1.14	5.73±0.51	11.53±0.88	7.73±0.51	18.43±0.98	58.88±7.29	84.63±1.11
Control group	4.58±0.71	7.50±0.96	5.63±0.93	11.58±1.22	5.58±0.68	11.40±1.06	7.60±0.67	14.38±1.15	58.63±6.98	80.00±1.52
t value	1.323	16.333	1.492	12.877	1.125	18.994	0.940	16.942	0.157	2.459
P value	0.190	0.000	0.140	0.000	0.264	0.000	0.350	0.000	0.876	0.016

The nursing satisfaction score in the observation group was (93.65±4.09) points, which was significantly higher than the (82.90±3.71) points in the control group; and the difference was statistically significant (*t*=12.325, *P*=0.000). The total incidence of complications in the observation group was lower than that in the control group; and the difference was statistically significant (χ^2^=5.591, *P*=0.018), (Table-V).

**Table-V:** Comparison of Incidence of Complications between the Two Groups [(%)].

Group	Headache	Emesis	Electrolyte disturbance	Re-hemorrhage	Hydrocephalus	Total
Observation group (n = 40)	1 (2.50)	1 (2.50)	0 (0.00)	2 (5.00)	1 (2.50)	5 (12.50)
Control group (n = 40)	4 (10.00)	3 (7.50)	2 (5.00)	3 (7.50)	2 (5.00)	14 (35.00)

## DISCUSSION

Studies have shown that,^8^,^9^ appropriate aerobic exercise, strength and neuromuscular training exercises can effectively help patients recover from SAH. In CNP, a well-structured rehabilitation exercise plan helps to promote patients’ recovery. On the one hand, CNP encourages patients to exercise in bed early after the operation, which not only contributes to later recovery, but also reduces the risk of lower limb vein thrombosis. On the other hand, CNP adds specific methods and steps of rehabilitation exercise nursing, and teaches patients how to exercise. The results of this study showed that the improvement of ADL in the observation group was significantly better than that in the control group, indicating that CNP can promote the post-operative recovery of patients’ self-care ability in daily life.

Studies have shown that^11^-^12^, the ADL score is associated with patients’ autonomous ability; and the ADL score is generally used to evaluate the degree of patients’ autonomous ability loss in nursing. Patients with scores lower than 20 have extremely serious dysfunction; 25-45 is classified as severe dysfunction; 50-70 is classified as moderate dysfunction; and 75-95 is classified as mild dysfunction. The score of 100 means complete self-care; when the score is lower than 35, patients are at high risk of discharge; 40-70 is classified as moderate risk of discharge; and more than 75 is classified as low risk of discharge. Patients with high ADL score, their autonomy is higher, and corresponding SS-QOL level can be improved. Some other studies showed that,^13^,^14^ CNP can improve the GCS score of patients with cerebral hemorrhage, improve their GOS score and cognitive function, and lower the incidence of POCD.

In this study, the opinions of experts and rehabilitation therapists were widely adopted when the CNP plan was designed: a lot of publicity and education and many ways of rehabilitation exercise were added in the CNP. The results of this study showed that the GCS, GOS, MMSE, SS-QOL and ADL scores of patients treated with CNP were higher than those in the control group, indicating that CNP has a positive effect on the prognosis of patients with SAH, and can improve the cognitive function, autonomy ability and QOL of patients.

Patients with SAH and their families are most concerned about the core issues of treatment program, treatment effect and patients’ final physiological condition.^15^ Therefore, during diagnosis and treatment, patients and their family members should be allowed to have an in-depth understanding of the treatment process of SAH, including treatment programs, nursing programs and rehabilitation exercises, so that patients and their family members can fully cooperate with the treatment and nursing, from passive acceptance to active cooperation, which not only improves the compliance of patients and their family members, but also improves the efficiency of nursing and rehabilitation exercises. This is conducive to the recovery of patients.^16^ The improvement of patients’ self-care ability not only improves their QOL, reduces the burden on patients’ families and reduces the use of medical resources to some extent.

A survey shows that,^17^ patients’ satisfaction is reflected in the nursing and treatment they receive. Reliable doctor-patient and nurse-patient relationships can make patients’ satisfaction at a high level; and good communication during the treatment can also improve patients’ satisfaction. Some other studies pointed out^18^,^19^ the provision of daily psychotherapy and allocation of medical staff are positively correlated to patients’ satisfaction. The patients and their family members in the observation group were informed of the precautions for operation, examination, nursing and treatment in strict accordance with the contents of CNP.

All operations were performed after patients and their family members had a certain understanding and consent. In this way, they could better cooperate with these operations. The results showed that CNP can provide timely nursing intervention before the occurrence of complications by means of steps and standard nursing measures; meanwhile, it can win the trust of patients and their family members, reduce patients’ anxiety and depression, and significantly improve patients’ satisfaction.

### Limitations

It includes small sample size, shorter period of follow-up, etc., which may produce a certain impact on the level of evidence of this study. Findings in this study are expected to be confirmed through further research based on a long-term follow-up with larger sample size.

## CONCLUSION

The CNP intervention in perioperative period of SAH patients has remarkable clinical effect. The development of personalized nursing plan according to patients’ condition can reduce the blindness of nursing, improve the pertinence and efficiency of nursing, help patients to recover as soon as possible, improve the QOL of patients and improve nursing satisfaction, and is worthy of clinical popularization.

### Authors’ Contributions:

**MG and NG:** Carried out the study, collected data, drafted the manuscript, are responsible and accountable for the accuracy and integrity of the work.

**YS:** Statistical analysis and participated in its design.

**NT:** Acquisition, analysis, or interpretation of data and drafting the manuscript.

All authors read and approved the final manuscript.
